# Seven psychiatric traits and the risk of increased carotid intima-media thickness: a Mendelian randomization study

**DOI:** 10.3389/fcvm.2024.1383032

**Published:** 2024-07-25

**Authors:** Kewan He, Jiajun Ying, Fangkun Yang, Teng Hu, Yuewu Du

**Affiliations:** ^1^ Department of Ultrasound, LiHuiLi Hospital, The Affiliated Hospital of Ningbo University, Ningbo, China; ^2^ Cardiology Center, Ningbo First Hospital, Ningbo University, Ningbo, China

**Keywords:** psychiatric trait, attention deficit/hyperactivity disorder, carotid intima-media thickness, arterial injury, Mendelian randomization

## Abstract

**Background:**

Numerous observational studies have suggested an association between psychiatric traits and carotid intima-media thickness (cIMT). However, whether these associations have a causal relationship remains unknown, largely due to issues of reverse causality and potential confounders. This study aims to elucidate the potential causal role of psychiatric traits in the risk of arterial injury as measured by cIMT.

**Methods:**

We utilized instrumental variables for attention deficit/hyperactivity disorder (ADHD, *n* = 226,534), bipolar disorder (*n* = 353,899), major depressive disorder (*n* = 142,646), post-traumatic stress disorder (*n* = 174,494), obsessive-compulsive disorder (*n* = 9,725), autism spectrum disorder (*n* = 173,773), and anxiety disease (*n* = 17,310), derived from the largest corresponding genome-wide association studies (GWAS). Summary statistics for cIMT associations were obtained from a meta-analysis combining GWAS data from the Cohorts for Heart and Aging Research in Genomic Epidemiology consortia (*n* = 71,128) and the UK Biobank study (*n* = 45,185). The inverse-variance weighted method served as the primary analytical tool, supplemented by additional statistical methods in the secondary analyses to corroborate the findings. Adjustments were made according to the Bonferroni correction threshold.

**Results:**

The Mendelian randomization analyses indicated a suggestive causal link between genetically predicted ADHD and cIMT (beta = 0.05; 95% confidence interval, 0.01–0.09; *p *= 0.018). Sensitivity analyses largely concurred with this finding. However, no significant associations were found between other psychiatric traits and cIMT.

**Conclusions:**

This study provides insights into the risk effect of ADHD on cIMT, suggesting that arteriopathy and potential associated complications should be considered during the treatment and monitoring of patients with ADHD.

## Introduction

Cardiovascular diseases (CVD) are the primary global mortality cause (1). Given that thickening of the arterial wall is an early indicator of subsequent plaque development, and because ultrasound can non-invasively measure carotid intima-media thickness (cIMT) (2, 3), cIMT is commonly utilized as a surrogate marker to study how early risk factors correlate with cardiovascular health.

Patients with psychiatric disorders have been observed to have an increased risk of premature all-cause mortality, especially from CVDs (4). Several studies have explored the association between mental characteristics and cIMT. However, these findings often suffer from inadequate adjustment for confounding factors and inconsistent definitions of mental health, thereby limiting causal inference. For instance, A meta-analysis of 19 prospective studies (4,490 cases vs. 27,583 controls) suggested that individuals with depressive symptoms had obviously thicker cIMT compared to the normal group without depressive symptoms (standard mean differences of 0.137; 95% CI, 0.047–0.227; *p *= 0.003) (5). However, the Young Finns Study did not observe a prospective relationship between depressive symptoms and subsequent cIMT (6). Furthermore, The Etude sur le Vieillissement Artériel study, a four-year prospective study investigation involving 726 middle-aged subjects, revealed a significant association between anxiety and an increase in cIMT in both males and females. Notably, this association remained independent of traditional cardiovascular risk factors (7). While a 2-year follow-up study of 518 postmenopausal women found that anxiety was associated with atherogenic lipid levels, but not with subclinical atherosclerosis (8). Overall, these findings suggest that the causal relationship between psychological traits and cIMT remains unclear.

Mendelian Randomization (MR) serves as a crucial method of instrumental variable analysis, improving the strength of causal inferences in observational epidemiological studies. MR analysis uses single nucleotide polymorphisms (SNPs) associated with specific exposures as instrumental variables (IVs) to assess if the relationship between an exposure and an outcome is causal (9). The underlying strength of MR lies in the random allocation of SNPs at conception, significantly reducing susceptibility to confounding biases. Additionally, since the genotype is not influenced by the phenotype, MR effectively minimizes confounding factors and the risk of reverse causality, thereby enhancing the validity of causal inferences in epidemiological research (10).

In recent years, a growing number of genome-wide association studies (GWAS) have been published (11–17), aiming to identify genetic risk loci associated with psychosocial factors. These studies lay a hopeful groundwork for evaluating the impact of mental health on cIMT from a genetic perspective. In this study, we utilize a two-sample MR design to explore the causality between psychiatric traits and arterial injury (18) as determined by cIMT.

## Method

### Study design

**Figure 1:**
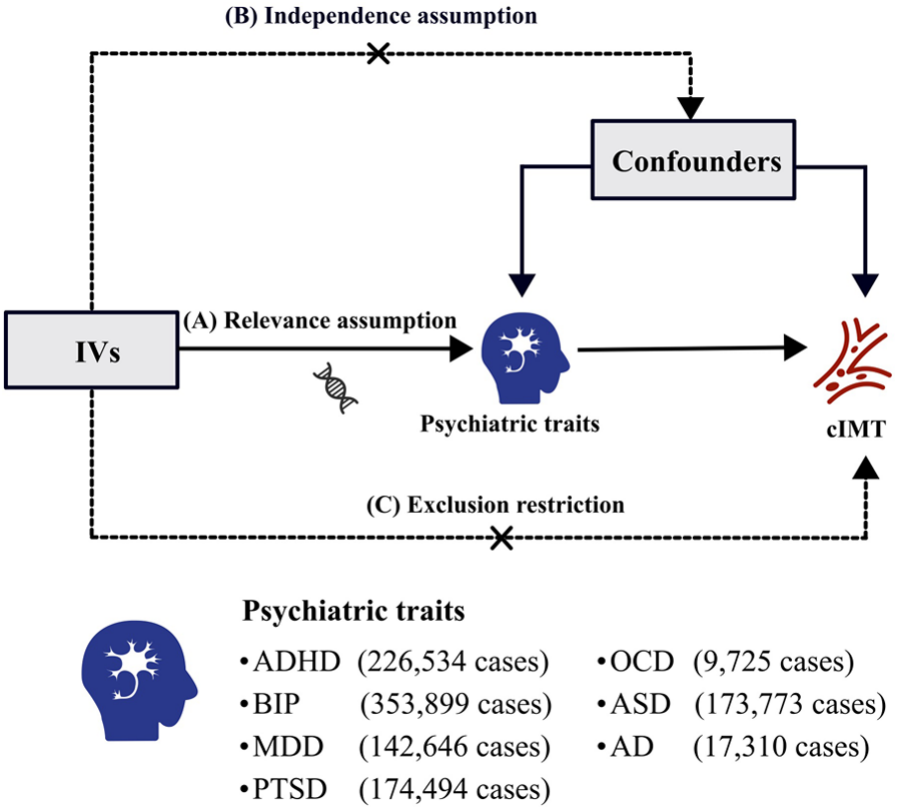
Three pivotal assumptions of MR analysis: (**A**) relevance assumption: the IVs must exhibit a strong correlation with the psychiatric traits; (**B**) independence assumption: IVs should not be associated with potential confounding factors; (**C**) exclusion restriction: IVs must influence the cIMT only via the psychiatric traits. IVs, instrumental variables; cIMT, carotid intima-media thickness; ADHD, attention deficit/hyperactivity disorder; BIP, bipolar disorder; MDD, major depressive disorder; PTSD, post-traumatic stress disorder; OCD, obsessive–compulsive disorder; ASD, autism spectrum disorder; AD, anxiety disease.

This study employed two-sample MR analyses, utilizing genetic summary-level genetic statistics from comprehensive and recent GWASs, to explore the causal relationship between seven psychiatric traits and cIMT. The IVs in this analysis were based on three rigorous assumptions (Figure 1): (a) Relevance assumption: IVs must demonstrate a strong correlation with the psychiatric traits; (b) Independence assumption: IVs should not be associated with potential confounding factors; (c) Exclusion restriction: IVs must influence cIMT exclusively through the psychiatric traits (19). All original studies adhered to ethical standards, including informed consent from participants and approval from relevant ethics committees.

### IVs selection

Estimates of SNP-exposure associations for seven psychiatric traits were sourced from the Psychiatric Genomics Consortium (PGC) (20), which includes attention deficit hyperactivity disorder (ADHD), bipolar disorder (BIP), major depressive disorder (MDD), post-traumatic stress disorder (PTSD), obsessive-compulsive disorder (OCD), autism spectrum disorder (ASD), and anxiety disorders (AD). As the largest global consortium in psychiatric research, PGC has significantly advanced the meta- and mega-analyses of genome-wide genetic data on psychiatric disorders (20). Detailed definitions for these seven psychiatric traits are outlined in Supplementary Table 1. SNPs strongly associated with exposure (BIP, ADHD) were identified at the genome-wide significance level (*p *< 5 × 10^−8^). For other psychiatric traits (MDD, PTSD, OCD, AD, and ASD), SNPs were selected using a less stringent threshold of 1 × 10^−5^. Linkage disequilibrium (LD) testing, based on the European 1,000 Genomes Project reference panel (*r*^2 ^< 0.01 and clump distance > 10,000 kb) (21), was conducted to ensure the independence of selected SNPs (21). In cases of LD, SNP with the higher *p*-value was excluded. Ultimately, 26 independent SNPs were identified for ADHD, 29 for BIP, 69 for MDD, 38 for PTSD, 25 for OCD, 58 for ASD, and 20 for AD. To reduce the risk of weak instrument bias, we applied an F statistic threshold of >10 to filter SNPs associated with the exposures (22).

### Data sources

The outcome-related SNPs for this study were sourced from a meta-analysis that combined data from the UK Biobank and the most extensive previous GWAS meta-analysis of cIMT conducted by the Cohorts for Heart and Aging Research in Genomic Epidemiology (CHARGE) consortia (Supplementary Table 2). This meta-analysis was performed to assess cIMTmax using the Multi-Trait Analysis of GWAS (MTAG) approach (23, 24). MTAG, a statistical instrument utilized for the analysis of aggregated data from multiple GWAS, implemented a generalized inverse variance-weighted (IVW) meta-analysis to mitigate biases stemming from sample overlap.

The CHARGE consortia's GWAS meta-analysis included 31 studies on cIMT, encompassing a total of 71,128 individuals (24), all of European descent. These participants were evaluated for cIMT parameters using high-resolution B-mode ultrasound. The UK Biobank, a prospective cohort based on the UK population, collected extensive genetic, physical, and health data from approximately 500,000 individuals aged 40–69 between 2006 and 2010. In the UK Biobank's genetic analysis of cIMT (involving 45,185 individuals), adjustments were made for gender, genotyping array, and the top 30 principal components to control for population stratification (23).

### Statistical analysis

Genetic IVs identified as psychiatric traits were queried for matching SNPs in the GWAS outcome. If specific SNPs were not present in the outcome data, proxy SNPs (*r*^2^ > 0.9) from the European population were used as substitutes, accessed via an online resource (https://ldlink.nci.nih.gov/?tab=ldproxy). The count of valid IVs for each exposure-outcome pairs is detailed in Table 1 (11–17).

**Table 1:** Data sources for SNPs.

Psychiatric traits	Population	Total sample size	Sample size (cases/controls)	Data souce	Number of significant associated SNPs
ADHD	Europeans	226,534	38,691/186,843	PGC	26
BIP	Europeans	353,899	40.463/31,3,436	PGC	28
MDD	Europeans	142,646	45,396/97,250	PGC	67^a^
PTSD	Europeans	174,494	23,185/151,309	PGC	32^a^
OCD	Europeans	9,725	2,688/7,037	PGC	23^a^
ASD	Europeans	173,773	16.539/157,234	PGC	56^a^
AD	Europeans	17,310	5,712/11,598	PGC	19^a^

SNPs, single nucleotide polymorphisms; ADHD, attention deficit/hyperactivity disorder; BIP, bipolar disorder; MDD, major depressive disorder; PTSD, post-traumatic stress disorder; OCD, obsessive-compulsive disorder; ASD, autism spectrum disorder; AD, anxiety disease.

^a^ The significance threshold for five psychiatric traits was *P*-value <1 × 10^−5^.

We employed IVW methods as the primary analytical approach to assess the effect of genetically predicted psychiatric traits on the risk of cIMT thickening (25). Using Wald estimation, we derived causal assessments for each SNP and generated corresponding standard errors through the delta method. These estimates were then integrated into a fixed-effects IVW meta-analysis to provide a comprehensive evaluation (26). Supplementary methods including the weighted median (WM), maximum likelihood (ML), MR-Egger regression, and leave-one-out sensitivity analysis were utilized to verify the robustness of our findings. The WM method allows for up to 50% of the IVs to be invalid while still yielding consistent estimates (27). The significance of the WM method value lies in its robustness to invalid instruments. Maximum likelihood, a standard approach for parameter estimation in probability distributions, maximizes the likelihood function to achieve lower standard errors (28). The MR-Egger method provides unbiased estimates even in the presence of horizontal pleiotropy (29). Additionally, leave-one-out sensitivity analysis was conducted to assess the stability of the results, sequentially excluding each SNP to test the reproducibility of the primary findings and ascertain if any single SNP significantly influenced the outcomes.

To detect potential horizontal pleiotropy, we used the MR-Egger intercept test (*p *< 0.05 indicating SNPs impact the outcome through pathways other than the exposure). The MR pleiotropy residual sum and outlier (MR-PRESSO) method was also employed for evaluating horizontal pleiotropy (30). This method, based on the IVW regression framework, identifies IVs with horizontal pleiotropy as outliers in the regression analysis. Specifically, MR-PRESSO detects SNPs with horizontal pleiotropy using a global test based on the leave-one-out procedure and an outlier test (31, 32). Cochran's Q statistics were used to examine heterogeneity among individual SNPs (33). If significant heterogeneity was identified (*p *< 0.05), a multiplicative random effects IVW analysis was performed. Following the Bonferroni correction standard, a *p*-value* *< 0.007 (0.05/7 tests) was considered indicative of a significant causal relationship between exposures and outcomes. *p*-values between 0.007 and 0.05 were deemed suggestive of associations.

All analyses were conducted using R version 4.3.1 and relevant R packages (i.e., “TwoSample MR” and “MR-PRESSO”) (21).

## Results

The IVs used for the MR analysis of seven psychiatric traits on cIMT are detailed in Supplementary Table 3–S9. The F-statistics of all IVs exceed the threshold of 10, indicating their strong predictive power for psychiatric traits in the MR analysis.

**Figure 2:**
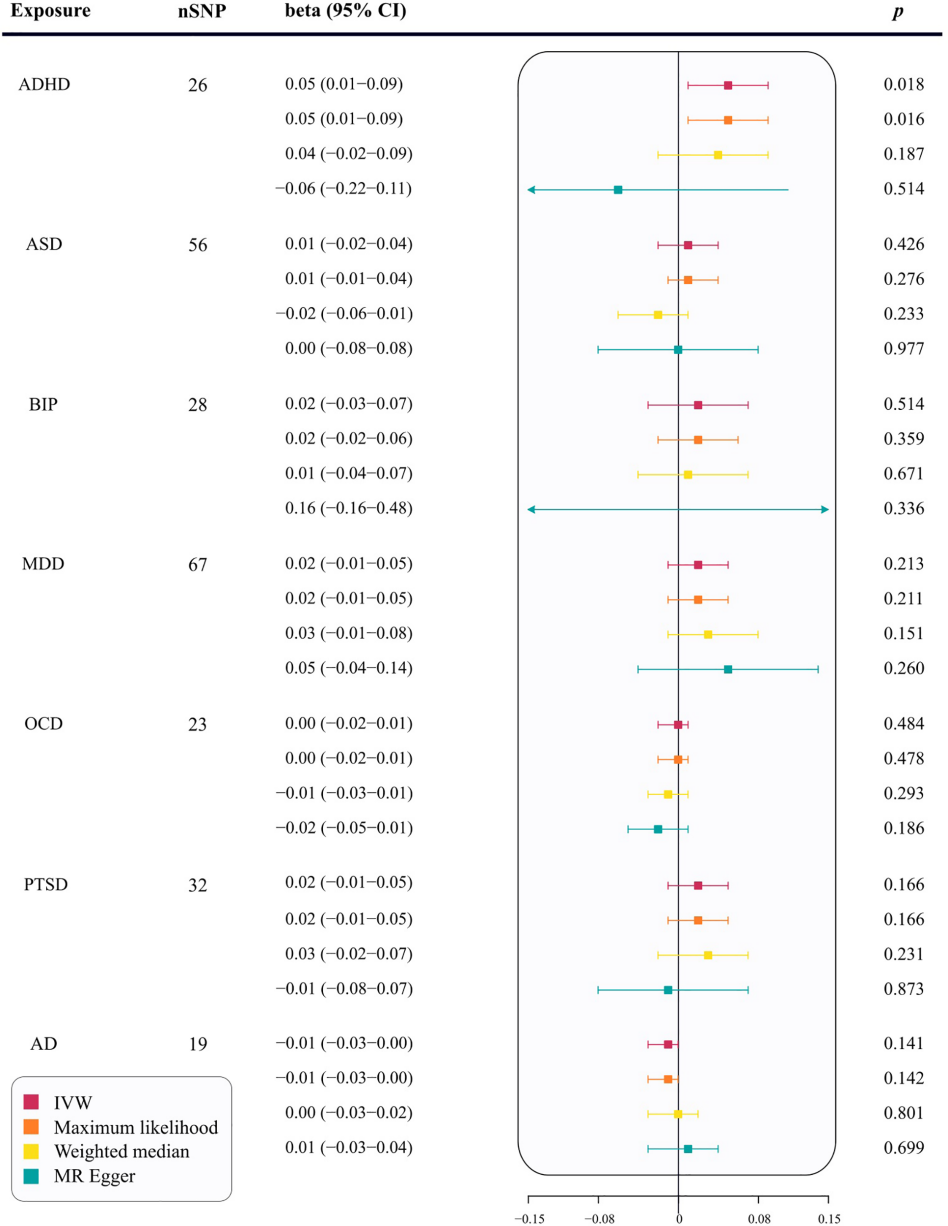
The correlation between seven psychiatric traits and cIMT in MR analysis. nSNP, number of single nucleotide polymorphisms; ADHD, attention deficit/hyperactivity disorder; BIP, bipolar disorder; MDD, major depressive disorder; PTSD, post-traumatic stress disorder; OCD, obsessive–compulsive disorder; ASD, autism spectrum disorder; AD, anxiety disease; SNPs, single-nucleotide polymorphisms; IVW, inverse-variance weighted; MR−Egger, Mendelian Randomization-Egger regression; CI, confidence interval; cIMT, carotid intima-media thickness; MR, Mendelian Randomization.

Using the IVW method, the results indicate a potential risk effect of ADHD on cIMT thickening, with a beta of 0.05 (95% CI = 0.01–0.09, *p *= 0.018). This suggests that with each unit increase in the log-odds of ADHD, the cIMT increases by 1.05 millimeters. Although not all methods used to evaluate the ADHD-cIMT association reached statistical significance, the direction of the association was consistent across most statistical models (Supplementary Figure 1). A stable relationship was also observed in the maximum likelihood analysis (beta = 0.05; 95% CI = 0.01–0.09; *p *= 0.016) (Figure 2). Sensitivity analysis of ADHD and cIMT using the Cochran's Q test, based on the IVW method, revealed no evidence of heterogeneity in the ADHD IVs (*p *= 0.054). Moreover, the intercept of the MR-Egger test was not statistically significant (*p *= 0.220), as well as the MR-PRESSO test did not find any outliers, indicating an absence of horizontal pleiotropy (Supplementary Table 10). The leave-one-out sensitivity analysis also demonstrated that no single instrumental SNP significantly affected the ADHD-cIMT causality (Supplementary Figure 1).

In the primary analysis, no causal relationships were found between ASD (beta = 0.01; 95% CI = −0.02–0.04; *p *= 0.426), BIP (beta = 0.02; 95% CI = −0.03–0.07; *p *= 0.514), MDD (beta = 0.02; 95% CI = −0.01–0.05; *p *= 0.213), OCD (beta = 0.00; 95% CI = −0.02–0.0; *p *= 0.484), PTSD (beta = 0.02; 95% CI = −0.01–0.05; *p *= 0.166), or AD (beta = −0.01; 95% CI = −0.03–0.00; *p *= 0.141) and cIMT thickening (Figure 2). Supplementary analyses, including maximum likelihood, weighted median, and MR-Egger, supported these findings (*p *> 0.05, Figure 2). The MR-Egger regression showed no evidence of horizontal pleiotropy (*p* for intercept < 0.05). MR-PRESSO analysis identified two outliers for ASD and one for BIP. However, the relationships remained stable after outlier exclusion, with no significant changes in estimates before and after outlier removal (*p* for distortion test >0.05) (Supplementary Table 10).

## Discussion

Utilizing the largest published GWAS, we applied a comprehensive framework to examine the genetic relationship between seven psychiatric traits and cIMT. Our analysis provides suggestive evidence for the potential causal effect of ADHD on the increased risk of cIMT thickening. Supplementary analyses also show directionally consistent association patterns across most statistical models.

Increased cIMT is a recognized marker of arterial injury and serves as an indicator of cardiovascular risk factors. Assessing a patient's cIMT, regardless of the presence of carotid plaques, can provide information on the potential occurrence of cardiovascular events in asymptomatic patients (34) and on cardiovascular outcomes in patients with known atherosclerotic disease (35). A meta-analysis incorporating 8 clinical studies with a total of over 37,000 patients and an average follow-up period of 5.5 years demonstrated a positive correlation between increased cIMT and the occurrence of cardiovascular events. This study suggested that for every 0.1 mm increase in cIMT, the future risk of myocardial infarction (MI) increases by ≤15% (36). Similarly, another meta-analysis concluded that combining the traditional Framingham Risk Score with cIMT may provide better predictive performance for stroke and MI (37). cIMT was also widely used as an efficacy endpoint for cardiovascular therapeutic drugs, such as statins, antihypertensives, and antidiabetics (38–41). Using cIMT measurements as a biomarker for atherosclerosis progression can facilitate the evaluation of treatment effectiveness before endpoints such as MI, stroke, and death occur. This approach can accelerate drug development and guide medication strategies earlier.

Previous studies had suggested that several psychiatric traits were associated with cIMT. In a study by Hakan et al. (42), 42 children with ADHD and 42 age- and sex- matched healthy controls were evaluated to assess the relationship between cIMT and ADHD symptom severity. Findings revealed significantly higher median cIMT values in the ADHD group compared to controls, with a notable correlation between cIMT and ADHD severity. Similar conclusions were echoed in subsequent research (43). However, these studies had limitations, including small sample sizes, making individual bias correction challenging, and limited follow-up periods, potentially leading to underestimation of incidence rates. Our research overcomes these constraints of traditional studies, and our findings on the ADHD-cIMT association are consistent with existing clinical research.

The link between ADHD and systemic inflammation has been extensively investigated. Nikola's (44) study found elevated markers of inflammation, including platelet distribution width, interleukins [interleukin -1β, interleukin-6 (IL-6)], Tumor Necrosis Factor-alpha (TNF-α), and the M1 proinflammatory profile, in adolescents with ADHD compared to healthy controls. This was further supported by a comprehensive meta-analysis showing generally higher IL-6 and lower TNF-α levels in individuals with ADHD (45). Chronic inflammation, known for its adverse impact on endothelial functions and its role in accelerating subclinical atherosclerosis via proinflammatory cytokines, is an emerging risk factor for CVD. It has also been independently linked to higher cIMT (46). Consequently, the collective evidence strongly suggests that chronic inflammation may be a key mechanism driving the development of arterial injury in ADHD populations.

Discrepancies exist in clinical research regarding the effects of depression on cIMT. In the Bogalusa study, Azad et al. conducted a cross-sectional study (47) enrolling 996 individuals aged 24–44 years to examine the association between depression symptoms and arterial injury, as determined by cIMT. The results indicated significant associations between depression scores and cIMT, particularly in individuals with a higher TC/HDL ratio. Similar findings were reported in a study focusing on police officers (48). However, the Young Finns study (6), involving 996 adults aged 30–45, found no association between cumulative depression index and cIMT, either before or after adjusting for traditional risk factors. Our findings suggest a lack of evidence for a causal relationship between MDD and cIMT, potentially elucidating contradictions observed in clinical trials.

Regarding anxiety and cIMT, evidence for a causal relationship is mixed. Itamar et al. (49) observed that individuals with higher symptoms of anxiety and/or depression had increased cIMT values, a finding echoed in subsequent studies (7, 50). However, a study targeting postmenopausal women in China found no significant association between perceived stress, anxiety, and cIMT (8), indicating that the causal relationship between anxiety and cIMT may vary by race and region. The disparities between these studies and ours could be attributed to potential biases and reverse causal relationships inherent in observational studies. Nevertheless, given the consistent results of the majority of prospective studies, the potential causal effects of anxiety on cIMT cannot be conclusively dismissed.

In this study, we utilized the largest GWAS meta-analysis to investigate the causal relationships between seven psychiatric traits and the risk of increased cIMT through MR analyses. Our research has several strengths. Firstly, we employed two-sample MR analyses to ascertain the causality between the seven psychiatric traits and cIMT (51). This method uses genetically assigned variations as IVs, effectively reducing confounding factors and reverse causality common in observational studies. Secondly, we sourced summary-level data from the most extensive GWAS dataset, enhancing the precision of SNP selection and the statistical robustness of our analyses. Finally, our robustness assessment, including multiple statistical models and leave-one-out analyses, strengthened the reliability of our primary findings.

Nevertheless, it is essential to recognize certain inherent limitations. Firstly, the GWAS dataset predominantly consisted of individuals of European ancestry, which may limit the generalizability of our findings to other ethnic groups. Additional studies are necessary to validate our results in non-European populations. Secondly, while we cannot entirely rule out the possibility of horizontal pleiotropy and its impact on our findings, it is noteworthy that our MR-PRESSO analysis did not indicate any significant evidence to this issue. Lastly, the lack of patient-level data, such as gender and socioeconomic status, constrained our capacity to explore the complex causal relationships between the seven psychiatric disorders and cIMT in relation to specific genders and social statuses. For instance, previous research has suggested that men with lower socioeconomic status might be more susceptible to increased cIMT compared to women (52).

## Conclusions

In summary, our study provides insights into the risk effect of ADHD on cIMT, primarily within the European population. When treating and monitoring patients with ADHD, consideration should be given to arteriopathy and potential related complications. Additional research is needed to elucidate the mechanisms and biological pathways linking psychiatric traits to cIMT.

## Data Availability

The original contributions presented in the study are included in the article/Supplementary Material, further inquiries can be directed to the corresponding author.
