# Supplementary material for: Seven psychiatric traits and the risk of increased carotid intima-media thickness: a Mendelian randomization study
Source: Front Cardiovasc Med. 2024 Jul 25;11:1383032. doi: 10.3389/fcvm.2024.1383032 (PMC11306041; doi:10.3389/fcvm.2024.1383032)
Supplement: Supplementary file 1 [file Datasheet1.docx]

**Supplementary figures**

**Supplement figure 1** Scatter plot, leave-one-out sensitivity analysis of the association between ADHD and the risk of cIMT thickening. (A) Scatter plot: Each dot represents a SNP; each line represents an estimate of the association between ADHD and the risk of cIMT thickening using corresponding methods. (B) Leave-one-out sensitivity analysis: The dot and bar represent the estimate and 95% CI after removing a specific SNP. ADHD, attention deficit/hyperactivity disorder; cIMT, carotid intima-media thickness; MR−Egger, Mendelian Randomization-Egger regression; CI, confidence interval; SNP, single-nucleotide polymorphisms.

**Index of Supplementary Tables**

Supplementary Table 1. Description of GWAS phenotype for each trait.

Supplementary Table 2. Detailed information on studies and datasets used for meta-analysis of cIMT-related SNPs

Supplementary Table 3. Twenty-six valid instrumental variables used for Mendelian randomization analysis of attention deficit hyperactivity disorder (Exposure) on cIMT (Outcome)

Supplementary Table 4. Twenty-eight valid instrumental variables used for Mendelian randomization analysis of bipolar disorder (Exposure) on cIMT (Outcome)

Supplementary Table 5. Sixty-seven valid instrumental variables used for Mendelian randomization analysis of major depressive disorder (Exposure) on cIMT (Outcome)

Supplementary Table 6. Thirty-two valid instrumental variables used for Mendelian randomization analysis of post-traumatic stress disorder (Exposure) on cIMT (Outcome)

Supplementary Table 7. Twenty-three valid instrumental variables used for Mendelian randomization analysis of obsessive–compulsive disorder (Exposure) on cIMT (Outcome)

Supplementary Table 8. Fifty-six valid instrumental variables used for Mendelian randomization analysis of autism spectrum disorder (Exposure) on cIMT (Outcome)

Supplementary Table 9. Nineteen valid instrumental variables used for Mendelian randomization analysis of anxiety disease (Exposure) on cIMT (Outcome)

Supplementary Table 10. Seven psychiatric traits and the risk of increased cIMT: a Mendelian randomization study

**Supplementary Table 1. Description of GWAS phenotype for each trait**

| **Trait** | **GWAS Phenotype Definition** |
| --- | --- |
| **ADHD** | Cases were diagnosed by psychiatrists at in- or out-patient clinics, predominantly the latter according to ICD10 |
| **BIP** | Cases met international consensus criteria (DSM-V or ICD-10) for a lifetime diagnosis of BIP |
| **MDD** | Cases met international consensus criteria (DSM-V or ICD-10) for a lifetime diagnosis of MDD |
| **PTSD** | cases assessment was based either on lifetime or current PTSD, and PTSD diagnosis was established using different versions of the DSM (DSM-III-R, DSM-IV, DSM-V) |
| **OCD** | Cases met international consensus criteria (DSM-IV) for a lifetime diagnosis of OCD |
| **ASD** | Cases were those met from either the Autism Diagnostic Interview-Revised or the Autism Diagnostic Observation Schedule domain scores |
| **AD** | Cases fit one of two definitions. First was self-reporting a lifetime professional diagnosis of an anxiety disorder. Second was meeting criteria for a likely lifetime diagnosis of DSM-IV generalized anxiety disorder |

GWAS, genome-wide association studies; ADHD, attention deficit hyperactivity disorder; BIP, bipolar disorder; MDD, major depressive disorder; PTSD, post-traumatic stress disorder; OCD, obsessive–compulsive disorder; ASD, autism spectrum disorder; AD, anxiety disease; DSM, diagnostic and statistical manual of mental disorders; ICD, international classification of diseases.

**Supplementary Table 2. Detailed information on studies and datasets used for meta-analysis of cIMT-related SNPs**

| **Data from** | **GWAS Reference** | **Population** | **Sample size** |
| --- | --- | --- | --- |
| UK Biobank | Yeung, M. W. et al. [21] | White participants (96.6%), Asian descent (1.4%), African descent (0.6%), a mixed descent (0.5%), and other/unknown ethnic background (0.6%) | 45,185 individuals |
| GWAS meta-analysis of cIMT conducted by the CHARGE consortia | Franceschini, N. et al. [22] | Europeans | 71,128 individuals |

cIMT, carotid intima-media thickness; SNPs, single-nucleotide polymorphisms; GWAS, genome-wide association studies; UK, the United Kingdom; CHARGE, Cohorts for Heart and Aging Research in Genomic Epidemiology.

**Supplementary Table 3. Twenty-six valid instrumental variables used for Mendelian randomization analysis of attention deficit hyperactivity disorder (Exposure) on cIMT (Outcome)**

| **SNP** | **Effect allele** | **Other allele** | **Beta** | **SE** | ***p*-value** |
| --- | --- | --- | --- | --- | --- |
| rs10875612 | C | T | -0.054 | 0.009 | 5.62E-09 |
| rs11255890 | C | A | 0.053 | 0.010 | 4.14E-08 |
| rs114142727 | C | G | 0.251 | 0.040 | 5.13E-10 |
| rs115111850 | A | G | -0.115 | 0.020 | 1.71E-08 |
| rs11596214 | G | A | 0.053 | 0.010 | 3.17E-08 |
| rs1162202 | C | T | 0.061 | 0.010 | 1.92E-09 |
| rs1438898 | A | C | 0.063 | 0.011 | 4.88E-09 |
| rs17576773 | C | T | 0.096 | 0.015 | 1.63E-10 |
| rs17718444 | C | T | 0.061 | 0.010 | 2.87E-09 |
| rs2025286 | A | C | -0.055 | 0.009 | 4.00E-09 |
| rs2582895 | C | A | 0.072 | 0.010 | 4.09E-14 |
| rs2886697 | G | A | 0.059 | 0.010 | 7.90E-10 |
| rs4916723 | A | C | -0.085 | 0.011 | 9.48E-15 |
| rs4925811 | T | G | -0.058 | 0.010 | 8.30E-09 |
| rs549845 | G | A | 0.079 | 0.010 | 9.03E-15 |
| rs6082363 | T | C | 0.070 | 0.010 | 4.38E-12 |
| rs6537401 | G | A | -0.057 | 0.010 | 1.40E-08 |
| rs704061 | T | C | -0.056 | 0.009 | 2.30E-09 |
| rs73145587 | A | T | 0.101 | 0.018 | 3.67E-08 |
| rs7506904 | G | A | -0.056 | 0.010 | 1.24E-08 |
| rs7613360 | C | T | -0.053 | 0.010 | 3.18E-08 |
| rs76284431 | T | A | -0.082 | 0.013 | 1.19E-09 |
| rs76857496 | C | A | 0.080 | 0.014 | 1.24E-08 |
| rs77960 | G | A | -0.073 | 0.010 | 2.46E-13 |
| rs7844069 | T | G | 0.055 | 0.010 | 6.74E-09 |
| rs9969232 | G | A | -0.068 | 0.010 | 9.98E-12 |

cIMT, carotid intima-media thickness; SNPs, single-nucleotide polymorphisms; SE, standard error.

**Supplementary Table 4. Twenty-eight valid instrumental variables used for Mendelian randomization analysis of bipolar disorder (Exposure) on cIMT (Outcome)**

| **SNP** | **Effect allele** | **Other allele** | **Beta** | **SE** | ***p*-value** |
| --- | --- | --- | --- | --- | --- |
| rs10737496 | C | T | 0.054 | 0.009 | 7.46E-09 |
| rs112481526 | A | G | -0.062 | 0.011 | 3.33E-09 |
| rs113779084 | G | A | -0.075 | 0.010 | 2.13E-13 |
| rs115694474 | T | A | 0.066 | 0.012 | 3.32E-08 |
| rs11764361 | A | G | 0.061 | 0.010 | 4.56E-09 |
| rs11870683 | T | A | 0.057 | 0.010 | 3.15E-08 |
| rs12154473 | A | G | -0.058 | 0.010 | 2.28E-09 |
| rs12575685 | G | A | -0.065 | 0.010 | 1.71E-10 |
| rs12932628 | G | T | -0.057 | 0.010 | 6.87E-09 |
| rs13044225 | A | G | -0.054 | 0.010 | 1.73E-08 |
| rs1487445 | C | T | -0.073 | 0.009 | 3.63E-15 |
| rs2273738 | C | T | -0.092 | 0.014 | 1.81E-11 |
| rs228768 | G | T | 0.065 | 0.010 | 2.45E-10 |
| rs2577831 | C | A | 0.066 | 0.009 | 1.44E-12 |
| rs2693698 | A | G | -0.053 | 0.009 | 2.61E-08 |
| rs28455634 | G | A | 0.063 | 0.010 | 3.04E-10 |
| rs28565152 | G | A | -0.066 | 0.011 | 3.29E-09 |
| rs35306827 | G | A | 0.066 | 0.011 | 4.06E-09 |
| rs35958438 | G | A | 0.065 | 0.012 | 2.72E-08 |
| rs4331993 | T | A | -0.055 | 0.010 | 2.30E-08 |
| rs4619651 | G | A | 0.066 | 0.010 | 5.17E-11 |
| rs4702 | G | A | 0.056 | 0.010 | 8.13E-09 |
| rs4840464 | T | A | -0.071 | 0.012 | 2.61E-09 |
| rs5758064 | T | C | 0.053 | 0.009 | 1.79E-08 |
| rs61846516 | C | G | -0.133 | 0.019 | 8.10E-12 |
| rs6992333 | A | G | -0.060 | 0.010 | 1.69E-09 |
| rs7201930 | T | C | -0.059 | 0.010 | 1.68E-08 |
| rs9834970 | T | C | -0.082 | 0.009 | 1.28E-18 |

cIMT, carotid intima-media thickness; SNPs, single-nucleotide polymorphisms; SE, standard error.

**Supplementary Table 5. Sixty-seven valid instrumental variables used for Mendelian randomization analysis of major depressive disorder (Exposure) on cIMT (Outcome)**

| **SNP** | **Effect allele** | **Other allele** | **Beta** | **SE** | ***p*-value** |
| --- | --- | --- | --- | --- | --- |
| rs10174534 | A | G | -0.044 | 0.009 | 1.49E-06 |
| rs1081458 | A | T | 0.060 | 0.012 | 5.20E-07 |
| rs10825942 | T | G | -0.048 | 0.009 | 3.50E-07 |
| rs11077049 | A | G | 0.142 | 0.030 | 2.56E-06 |
| rs11235880 | A | C | 0.050 | 0.011 | 9.36E-06 |
| rs114923224 | T | C | 0.078 | 0.018 | 7.11E-06 |
| rs117141160 | A | G | 0.268 | 0.057 | 2.68E-06 |
| rs11725076 | A | T | -0.052 | 0.012 | 7.66E-06 |
| rs1219935 | T | C | 0.074 | 0.016 | 2.00E-06 |
| rs12200766 | A | G | 0.048 | 0.010 | 3.71E-06 |
| rs12500670 | T | C | -0.067 | 0.015 | 6.57E-06 |
| rs12552 | A | G | 0.043 | 0.009 | 2.19E-06 |
| rs1256112 | T | C | 0.044 | 0.009 | 9.44E-07 |
| rs12606521 | A | G | 0.054 | 0.012 | 3.15E-06 |
| rs1361500 | T | C | -0.043 | 0.009 | 5.01E-06 |
| rs138203289 | C | G | -0.193 | 0.044 | 9.10E-06 |
| rs138690408 | A | T | 0.178 | 0.040 | 9.80E-06 |
| rs141811146 | A | G | 0.224 | 0.050 | 6.91E-06 |
| rs144895331 | T | C | -0.089 | 0.019 | 4.37E-06 |
| rs1468520 | A | G | -0.054 | 0.012 | 6.49E-06 |
| rs1491473 | T | C | 0.110 | 0.023 | 9.83E-07 |
| rs150888111 | A | C | -0.225 | 0.051 | 9.28E-06 |
| rs1548352 | A | T | 0.041 | 0.009 | 8.77E-06 |
| rs17043773 | T | C | 0.046 | 0.010 | 4.51E-06 |
| rs17499892 | A | C | -0.050 | 0.009 | 6.64E-08 |
| rs1888286 | T | C | 0.048 | 0.010 | 1.07E-06 |
| rs1950829 | A | G | 0.054 | 0.009 | 8.51E-10 |
| rs2012697 | T | C | -0.048 | 0.009 | 1.14E-07 |
| rs2060886 | T | C | -0.046 | 0.009 | 3.86E-07 |
| rs2084080 | A | G | -0.056 | 0.012 | 3.41E-06 |
| rs2451828 | T | C | 0.159 | 0.031 | 2.07E-07 |
| rs2784590 | T | C | 0.051 | 0.011 | 2.96E-06 |
| rs281265 | A | C | -0.046 | 0.009 | 8.73E-07 |
| rs34318462 | C | G | 0.049 | 0.010 | 3.04E-06 |
| rs34382743 | T | C | 0.046 | 0.009 | 3.34E-07 |
| rs35448533 | T | C | -0.054 | 0.012 | 7.73E-06 |
| rs35654319 | T | C | -0.067 | 0.015 | 8.63E-06 |
| rs3793406 | C | G | -0.045 | 0.010 | 5.04E-06 |
| rs3923290 | A | G | -0.043 | 0.010 | 8.17E-06 |
| rs4141983 | T | C | 0.043 | 0.010 | 7.42E-06 |
| rs4776768 | T | C | -0.050 | 0.010 | 7.37E-07 |
| rs4811079 | C | G | 0.049 | 0.009 | 9.30E-08 |
| rs4852123 | T | C | 0.054 | 0.011 | 2.01E-06 |
| rs55825782 | T | C | 0.044 | 0.010 | 5.06E-06 |
| rs56308316 | T | C | -0.099 | 0.022 | 4.61E-06 |
| rs586275 | A | G | -0.059 | 0.012 | 8.44E-07 |
| rs6030655 | T | C | -0.071 | 0.016 | 8.61E-06 |
| rs60747160 | T | G | 0.045 | 0.009 | 1.67E-06 |
| rs615747 | A | T | 0.079 | 0.017 | 3.91E-06 |
| rs62006671 | A | G | 0.071 | 0.015 | 1.37E-06 |
| rs62284515 | T | C | -0.055 | 0.012 | 3.93E-06 |
| rs6511757 | A | G | -0.213 | 0.048 | 9.80E-06 |
| rs6689226 | T | C | 0.050 | 0.009 | 8.55E-08 |
| rs6832890 | C | G | -0.060 | 0.011 | 1.09E-07 |
| rs7127852 | A | T | -0.056 | 0.012 | 2.70E-06 |
| rs71521437 | C | G | 0.101 | 0.022 | 4.44E-06 |
| rs73186196 | T | G | 0.159 | 0.036 | 9.34E-06 |
| rs74336531 | A | C | 0.096 | 0.020 | 2.00E-06 |
| rs74837665 | T | C | 0.075 | 0.015 | 1.10E-06 |
| rs76025409 | C | G | 0.057 | 0.010 | 2.45E-09 |
| rs76837483 | T | G | 0.097 | 0.020 | 1.73E-06 |
| rs77198573 | C | G | -0.046 | 0.010 | 3.93E-06 |
| rs78676209 | C | G | -0.104 | 0.020 | 3.20E-07 |
| rs875270 | T | C | -0.059 | 0.013 | 2.25E-06 |
| rs9327211 | T | C | 0.042 | 0.009 | 5.77E-06 |
| rs9655918 | A | G | -0.044 | 0.010 | 5.72E-06 |
| rs9926691 | A | G | -0.044 | 0.010 | 5.83E-06 |

cIMT, carotid intima-media thickness; SNPs, single-nucleotide polymorphisms; SE, standard error.

**Supplementary Table 6. Thirty-two valid instrumental variables used for Mendelian randomization analysis of post-traumatic stress disorder (Exposure) on cIMT (Outcome)**

| **SNP** | **Effect allele** | **Other allele** | **Beta** | **SE** | ***p*-value** |
| --- | --- | --- | --- | --- | --- |
| rs1136201 | A | G | -0.080 | 0.017 | 2.34E-06 |
| rs114514029 | T | G | -0.204 | 0.045 | 5.84E-06 |
| rs117405401 | A | G | 0.256 | 0.055 | 3.69E-06 |
| rs117453028 | T | C | 0.286 | 0.063 | 5.81E-06 |
| rs1268149 | A | G | 0.380 | 0.083 | 4.53E-06 |
| rs12706983 | T | G | -0.090 | 0.019 | 3.01E-06 |
| rs1363508 | A | G | -0.072 | 0.016 | 7.39E-06 |
| rs139591016 | T | C | -0.249 | 0.053 | 2.86E-06 |
| rs140281028 | A | C | 0.170 | 0.038 | 9.07E-06 |
| rs140928208 | A | G | 0.447 | 0.093 | 1.46E-06 |
| rs1444764 | A | G | 0.078 | 0.016 | 1.90E-06 |
| rs146676625 | A | G | 0.233 | 0.052 | 6.94E-06 |
| rs149509653 | A | G | 0.090 | 0.019 | 1.74E-06 |
| rs17041470 | A | G | -0.298 | 0.064 | 3.66E-06 |
| rs17108326 | A | G | 0.106 | 0.021 | 3.91E-07 |
| rs184613502 | A | G | 0.295 | 0.066 | 7.36E-06 |
| rs2163050 | A | G | 0.100 | 0.021 | 2.12E-06 |
| rs2293995 | T | C | 0.168 | 0.037 | 7.62E-06 |
| rs36127550 | T | G | -0.103 | 0.020 | 4.63E-07 |
| rs57753395 | A | G | 0.376 | 0.071 | 9.13E-08 |
| rs62583551 | A | G | -0.097 | 0.020 | 1.22E-06 |
| rs72657988 | T | G | 0.147 | 0.033 | 6.61E-06 |
| rs72914051 | T | G | -0.133 | 0.029 | 5.61E-06 |
| rs73154700 | A | G | 0.126 | 0.027 | 3.45E-06 |
| rs73157523 | T | C | -0.086 | 0.020 | 8.65E-06 |
| rs74901405 | T | G | 0.117 | 0.026 | 6.81E-06 |
| rs763753 | A | G | -0.114 | 0.022 | 2.43E-07 |
| rs77537694 | A | G | 0.130 | 0.028 | 3.25E-06 |
| rs7807190 | A | G | 0.083 | 0.018 | 4.04E-06 |
| rs78608260 | A | G | -0.321 | 0.069 | 3.71E-06 |
| rs79317376 | T | G | 0.325 | 0.073 | 8.39E-06 |
| rs80074987 | T | G | 0.238 | 0.051 | 2.64E-06 |

cIMT, carotid intima-media thickness; SNPs, single-nucleotide polymorphisms; SE, standard error.

**Supplementary Table 7. twenty-three valid instrumental variables used for Mendelian randomization analysis of obsessive–compulsive disorder (Exposure) on cIMT (Outcome)**

| **SNP** | **Effect allele** | **Other allele** | **Beta** | **SE** | ***p*-value** |
| --- | --- | --- | --- | --- | --- |
| rs1030757 | A | C | -0.165 | 0.034 | 1.09E-06 |
| rs10773765 | T | C | 0.184 | 0.040 | 5.19E-06 |
| rs116347760 | A | T | 0.630 | 0.134 | 2.39E-06 |
| rs117310268 | T | C | 0.449 | 0.097 | 3.31E-06 |
| rs12568997 | A | G | -0.293 | 0.058 | 4.23E-07 |
| rs13141765 | T | C | -0.266 | 0.056 | 1.86E-06 |
| rs138445568 | A | T | -0.928 | 0.207 | 7.71E-06 |
| rs139286049 | A | G | -0.601 | 0.135 | 7.82E-06 |
| rs1652783 | A | G | -0.269 | 0.059 | 6.09E-06 |
| rs28599745 | A | G | -0.363 | 0.080 | 6.00E-06 |
| rs3097331 | T | C | 0.157 | 0.035 | 7.46E-06 |
| rs35894340 | A | G | -0.175 | 0.039 | 8.45E-06 |
| rs4733767 | A | G | 0.194 | 0.039 | 7.10E-07 |
| rs55687617 | A | G | -0.270 | 0.058 | 2.67E-06 |
| rs56025909 | T | C | 0.412 | 0.092 | 7.19E-06 |
| rs56343802 | A | T | -0.170 | 0.037 | 3.97E-06 |
| rs639560 | T | C | -0.416 | 0.088 | 2.23E-06 |
| rs72781967 | T | C | -0.167 | 0.035 | 2.43E-06 |
| rs72783425 | A | C | 0.340 | 0.073 | 3.53E-06 |
| rs75740353 | A | G | -0.433 | 0.098 | 9.75E-06 |
| rs77885126 | T | C | -0.603 | 0.131 | 4.38E-06 |
| rs9544927 | A | G | 0.193 | 0.043 | 5.65E-06 |
| rs9952159 | T | C | 0.182 | 0.040 | 4.21E-06 |

cIMT, carotid intima-media thickness; SNPs, single-nucleotide polymorphisms; SE, standard error.

**Supplementary Table 8. Fifty-six valid instrumental variables used for Mendelian randomization analysis of autism spectrum disorder (Exposure) on cIMT (Outcome)**

| **SNP** | **Effect allele** | **Other allele** | **Beta** | **SE** | ***p*-value** |
| --- | --- | --- | --- | --- | --- |
| rs10099100 | C | G | 0.08 | 0.01 | 1.07E-08 |
| rs10110094 | A | G | 0.09 | 0.02 | 2.05E-06 |
| rs11185408 | A | G | -0.07 | 0.01 | 6.98E-07 |
| rs111931861 | A | G | -0.22 | 0.04 | 1.12E-07 |
| rs112635299 | T | G | 0.22 | 0.04 | 3.04E-07 |
| rs113003385 | A | G | 0.13 | 0.03 | 6.06E-06 |
| rs113764414 | A | G | 0.07 | 0.02 | 9.43E-06 |
| rs115833252 | T | C | 0.23 | 0.05 | 8.74E-06 |
| rs116346488 | A | G | 0.14 | 0.03 | 7.58E-06 |
| rs116977567 | T | G | -0.20 | 0.04 | 5.04E-06 |
| rs11787216 | T | C | -0.07 | 0.01 | 2.59E-06 |
| rs13201465 | A | G | -0.28 | 0.06 | 6.42E-06 |
| rs141319505 | A | G | 0.29 | 0.06 | 1.88E-06 |
| rs141455452 | T | G | 0.08 | 0.02 | 8.94E-07 |
| rs144911765 | T | C | -0.19 | 0.04 | 2.36E-06 |
| rs1452075 | T | C | 0.08 | 0.02 | 2.07E-07 |
| rs148587110 | T | C | -0.33 | 0.07 | 7.40E-06 |
| rs149923766 | T | G | -0.24 | 0.05 | 9.61E-07 |
| rs1522603 | T | C | 0.06 | 0.01 | 5.34E-06 |
| rs16879023 | A | G | -0.10 | 0.02 | 1.77E-06 |
| rs16933101 | A | G | -0.15 | 0.03 | 9.42E-06 |
| rs17517971 | A | G | 0.14 | 0.03 | 9.62E-06 |
| rs201179706 | T | C | -0.07 | 0.02 | 9.82E-06 |
| rs2224274 | T | C | 0.07 | 0.01 | 2.86E-07 |
| rs2391769 | A | G | -0.08 | 0.01 | 1.14E-07 |
| rs28729902 | A | G | -0.08 | 0.02 | 2.35E-06 |
| rs292441 | A | G | -0.07 | 0.01 | 1.12E-06 |
| rs325485 | A | G | 0.07 | 0.01 | 3.25E-07 |
| rs34509057 | A | G | 0.07 | 0.02 | 9.73E-06 |
| rs35404050 | T | C | 0.08 | 0.02 | 1.61E-06 |
| rs41363353 | C | G | -0.08 | 0.02 | 8.61E-06 |
| rs45595836 | T | C | 0.14 | 0.03 | 3.13E-07 |
| rs4609618 | A | C | -0.06 | 0.01 | 9.34E-06 |
| rs4750990 | T | C | -0.07 | 0.01 | 1.37E-06 |
| rs4916723 | A | C | -0.07 | 0.01 | 1.92E-06 |
| rs529507 | A | G | -0.09 | 0.02 | 5.76E-06 |
| rs597040 | C | G | 0.26 | 0.06 | 7.37E-06 |
| rs6430841 | A | G | 0.08 | 0.02 | 6.40E-06 |
| rs644552 | A | G | 0.16 | 0.03 | 4.21E-06 |
| rs6692705 | A | G | 0.07 | 0.01 | 3.26E-06 |
| rs6701243 | A | C | 0.07 | 0.01 | 3.07E-07 |
| rs684502 | T | G | -0.06 | 0.01 | 9.71E-06 |
| rs72934503 | A | G | -0.07 | 0.01 | 5.89E-07 |
| rs740883 | A | T | -0.11 | 0.02 | 1.69E-06 |
| rs7578456 | A | G | 0.06 | 0.01 | 6.53E-06 |
| rs76397219 | A | G | -0.14 | 0.03 | 3.57E-06 |
| rs77691144 | T | C | -0.21 | 0.04 | 1.91E-06 |
| rs7783557 | T | C | 0.07 | 0.01 | 4.36E-06 |
| rs78058104 | A | G | 0.19 | 0.04 | 2.22E-06 |
| rs78298487 | T | G | -0.26 | 0.06 | 9.01E-06 |
| rs78653484 | T | C | -0.18 | 0.04 | 4.68E-06 |
| rs78827416 | A | G | 0.13 | 0.03 | 9.00E-07 |
| rs79940520 | A | G | -0.10 | 0.02 | 4.26E-06 |
| rs910805 | A | G | -0.10 | 0.02 | 2.04E-09 |
| rs9366877 | A | G | 0.07 | 0.01 | 9.05E-07 |
| rs9389208 | T | C | 0.07 | 0.01 | 3.12E-06 |

cIMT, carotid intima-media thickness; SNPs, single-nucleotide polymorphisms; SE, standard error.

**Supplementary Table 9. Nineteen valid instrumental variables used for Mendelian randomization analysis of anxiety disease (Exposure) on cIMT (Outcome)**

| **SNP** | **Effect allele** | **Other allele** | **Beta** | **SE** | ***p*-value** |
| --- | --- | --- | --- | --- | --- |
| rs1067330 | A | G | 0.022 | 0.005 | 0.268 |
| rs10883597 | T | C | -0.033 | 0.007 | 0.403 |
| rs10956359 | T | C | 0.023 | 0.005 | 0.702 |
| rs112311059 | T | C | 0.182 | 0.039 | 0.942 |
| rs116274579 | A | G | 0.141 | 0.031 | 0.055 |
| rs11998109 | T | C | -0.020 | 0.004 | 0.545 |
| rs13340324 | A | C | 0.023 | 0.005 | 0.497 |
| rs17823065 | T | C | -0.049 | 0.011 | 0.898 |
| rs4724582 | A | G | -0.020 | 0.005 | 0.533 |
| rs6068466 | A | G | 0.025 | 0.006 | 0.248 |
| rs72850179 | A | G | -0.055 | 0.012 | 0.788 |
| rs75157826 | T | G | 0.049 | 0.011 | 0.060 |
| rs79310980 | T | C | -0.064 | 0.014 | 0.948 |
| rs9949003 | A | G | -0.029 | 0.007 | 0.860 |

cIMT, carotid intima-media thickness; SNPs, single-nucleotide polymorphisms; SE, standard error.

**Supplementary Table 10. Seven psychiatric traits and the risk of increased cIMT: a Mendelian randomization study**

| **Exposure** | **Used SNPs** | **Q** | ***p*_Q_** | **MR-Egger** | | |
| --- | --- | --- | --- | --- | --- | --- |
|  |  |  |  | **Beta** | **95% CI** | ***p*** |
| ADHD | 26 | 23 | 0.504 | 0.95 | 0.80,1.11 | 0.514 |
| ASD | 56 | 103 | 6.83E-05 | 1.00 | 0.92,1.09 | 0.977 |
| BIP | 28 | 51 | 0.003 | 1.17 | 0.85,1.61 | 0.336 |
| MDD | 67 | 62 | 0.578 | 1.05 | 0.96,1.15 | 0.260 |
| OCD | 23 | 17 | 0.685 | 0.98 | 0.95,1.01 | 0.186 |
| PTSD | 32 | 38 | 0.138 | 0.99 | 0.92,1.07 | 0.873 |
| AD | 19 | 17 | 0.154 | 1.01 | 0.97,1.05 | 0.699 |
| **Exposure** | ***p*_pleiotropy_^a^** | | ***p*_distortion test_^b^** | **MR-PRESSO^c^** | | |
|  |  |  |  | **Beta** | **95% CI** | ***p*** |
| ADHD | 0.221 | | - | 1.05 | 1.01,1.09 | 0.026 |
| ASD | 0.762 | | 0.173 | 0.99 | 0.97,1.02 | 0.606 |
| BIP | 0.384 | | 0.637 | 1.03 | 0.99,1.08 | 0.158 |
| MDD | 0.462 | | - | 1.02 | 0.98,1.05 | 0.405 |
| OCD | 0.249 | | - | 1.00 | 0.99,1.01 | 0.731 |
| PTSD | 0.432 | | - | 1.02 | 0.99,1.05 | 0.305 |
| AD | 0.927 | | - | 0.98 | 0.90,1.06 | 0.586 |

cIMT, carotid intima-media thickness; SNPs, single-nucleotide polymorphisms; ADHD, attention deficit hyperactivity disorder; BIP, bipolar disorder; MDD, major depressive disorder; PTSD, post-traumatic stress disorder; OCD, obsessive–compulsive disorder; ASD, autism spectrum disorder; AD, anxiety disease; MR−Egger, Mendelian Randomization-Egger regression; OR, odds ratio; CI, confidence interval; MR-PRESSO, mendelian randomization pleiotropy residual sum and outlier.

^a^ ***p***-values for pleiotropy were ***p***-values for MR-Egger intercept test and a ***p***-value<0.05 indicates a statistically significant pleiotropic effect

^b^ ***p***-values for distortion were obtained from MR-PRESSO test and a ***p***-value<0.05 indicates a statistically significant difference between estimates before and after outlier removal. ***p*** of distortion test was not available for the analysis of ADHD, MDD, OCD, PTSD, and AD, as no outliers were detected

^c^There were 2 outliers detected in MR-PRESSO analysis of ASD, and 1 in BIP
