# Supplementary figures and images for: Seven psychiatric traits and the risk of increased carotid intima-media thickness: a Mendelian randomization study
Source: Front Cardiovasc Med. 2024 Jul 25;11:1383032. doi: 10.3389/fcvm.2024.1383032 (PMC11306041; doi:10.3389/fcvm.2024.1383032)

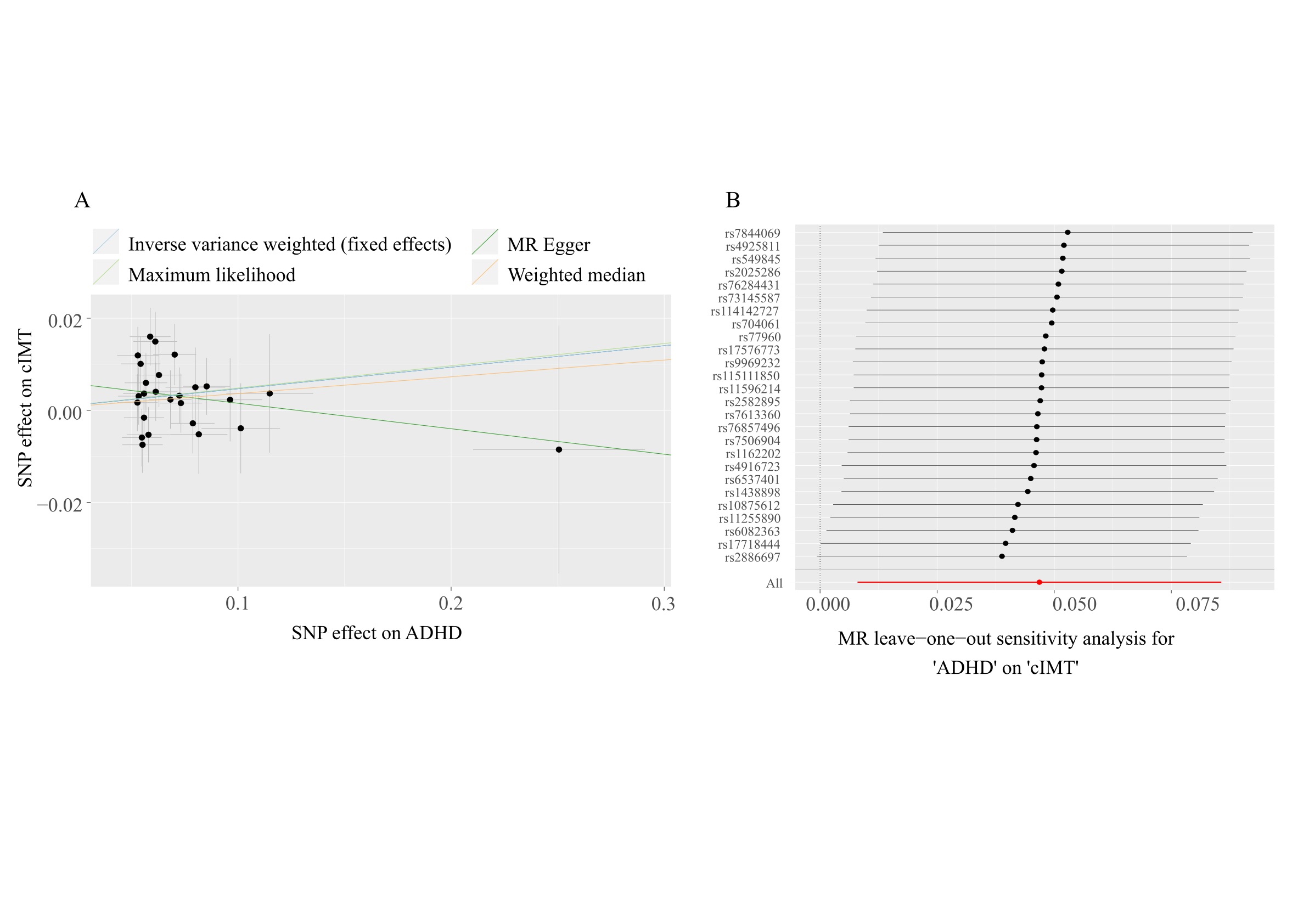

Supplement: Supplementary file 2 [file Image1.jpeg]
